# Machine learning models-based on integration of next-generation sequencing testing and tumor cell sizes improve subtype classification of mature B-cell neoplasms

**DOI:** 10.3389/fonc.2023.1160383

**Published:** 2023-08-03

**Authors:** Yafei Mu, Yuxin Chen, Yuhuan Meng, Tao Chen, Xijie Fan, Jiecheng Yuan, Junwei Lin, Jianhua Pan, Guibin Li, Jinghua Feng, Kaiyuan Diao, Yinghua Li, Shihui Yu, Lingling Liu

**Affiliations:** ^1^ Department of Hematology, The Third Affiliated Hospital of Sun Yat‐sen University and Sun Yat‐sen Institute of Hematology, Guangzhou, China; ^2^ KingMed School of Laboratory Medicine, Guangzhou Medical University, Guangzhou, China; ^3^ Guangzhou KingMed Transformative Medicine Institute Co., Ltd., Guangzhou, China; ^4^ Guangzhou KingMed Center for Clinical Laboratory Co., Ltd., Guangzhou, China; ^5^ Guangzhou KingMed Diagnostics Group Co., Ltd., Guangzhou, China

**Keywords:** mature B-cell neoplasms (MBNs), pathological diagnosis, next-generation sequencing (NGS), machine learning (ML), subtype classification

## Abstract

**Background:**

Next-generation sequencing (NGS) panels for mature B-cell neoplasms (MBNs) are widely applied clinically but have yet to be routinely used in a manner that is suitable for subtype differential diagnosis. This study retrospectively investigated newly diagnosed cases of MBNs from our laboratory to investigate mutation landscapes in Chinese patients with MBNs and to combine mutational information and machine learning (ML) into clinical applications for MBNs, especially for subtype classification.

**Methods:**

Samples from the Catalogue Of Somatic Mutations In Cancer (COSMIC) database were collected for ML model construction and cases from our laboratory were used for ML model validation. Five repeats of 10-fold cross-validation Random Forest algorithm was used for ML model construction. Mutation detection was performed by NGS and tumor cell size was confirmed by cell morphology and/or flow cytometry in our laboratory.

**Results:**

Totally 849 newly diagnosed MBN cases from our laboratory were retrospectively identified and included in mutational landscape analyses. Patterns of gene mutations in a variety of MBN subtypes were found, important to investigate tumorigenesis in MBNs. A long list of novel mutations was revealed, valuable to both functional studies and clinical applications. By combining gene mutation information revealed by NGS and ML, we established ML models that provide valuable information for MBN subtype classification. In total, 8895 cases of 8 subtypes of MBNs in the COSMIC database were collected and utilized for ML model construction, and the models were validated on the 849 MBN cases from our laboratory. A series of ML models was constructed in this study, and the most efficient model, with an accuracy of 0.87, was based on integration of NGS testing and tumor cell sizes.

**Conclusions:**

The ML models were of great significance in the differential diagnosis of all cases and different MBN subtypes. Additionally, using NGS results to assist in subtype classification of MBNs by method of ML has positive clinical potential.

## Introduction

Mature B-cell neoplasms (MBNs) are induced by monoclonal proliferation and expansion of mature B-cell original lymphocytes (1, 2). According to the 2016 revision of the World Health Organization classification of lymphoid neoplasms (2), laboratory diagnosis of MBNs relies on morphology immunology, cytogenetics, and molecular biology (MICM classification system). While morphology is considered to be the major feature for the diagnosis of MBNs, immunophenotype, cytogenetics, and molecular biology are more informative for MBN pathological subtype classification, precision therapy, and prognostic evaluation (2, 3). The significance of genetic testing has been further emphasized by the identification of an increasing number of recurrent gene abnormalities in MBNs through the widespread application of next-generation sequencing (NGS) techniques (4, 5).

Different pathological subtypes of MBNs have been found to have heterogeneous mutation landscapes (2, 4). The most recurrently mutated genes identified in chronic lymphocytic leukemia/small lymphocytic lymphoma (CLL/SLL) are *TP53*, *NOTCH1*, *SF3B1*, and *BIRC3* (6–8). In diffuse large B-cell lymphoma (DLBCL), *EZH2* and *GNA13* variants are observed exclusively in the germinal center B-cell subtype, whereas *CARD11*, *MYD88*, and *CD79B* variants are characteristic of the activated B-cell subtype (9, 10). In follicular lymphoma (FL), variants of *EZH2*, *ARID1A*, *MEF2B*, *EP300*, *FOX01*, *CREBBP*, and *CARD11* have been reported to be associated with prognosis, with recent addition of recurrent *STAT6* and *MAP2K1* variants in the list (11, 12). Abnormalities in *ATM*, *TP53*, and *CCND1* have been reported in mantle cell lymphoma (MCL) (13–15), while *MYD88* (especially *MYD88* L265P) and *CXCR4* mutations have been identified in lymphoplasmacytic lymphoma/Waldenstrom macroglobulinemia (LPL/WM) (16, 17). Thus, integration of recent molecular findings into MBN subtype classification is very encouraging, especially considering cases that are difficult to subcategorize based on the current MICM classification system.

The establishment and application of machine learning (ML) have been found to facilitate the development of new tools for integrating data from a variety of platforms with highly accurate and detailed assessments for predicting disease prognosis (18–20). In this study, we retrospectively obtained the mutation landscape of MBNs, established ML models by integrating mutation data and other laboratory parameters, and then validated these models in the prediction of MBN subtype classifications.

## Materials and methods

### Patients and specimens

From January 1^st^, 2018, to December 31^st^, 2019, out of all retrospective cases in our laboratory with morphological examination and immunophenotypical testing routinely being used in clinical practice while genetic and genomic tests being performed occasionally according to referring doctors’ orders, 849 cases of newly diagnosed MBNs were identified and included for further analyses. Each patient was diagnosed and classified according to the 2016 revision of the World Health Organization classification of lymphoid neoplasms (MICM classification system). In addition, two diagnostic groups were defined in this study based on the MICM system: 1) initial diagnosis was primarily based on morphologic and immunophenotypic information, and 2) comprehensive diagnosis was based on test results from multiple platforms, including morphology, immunophenotype, NGS, and some other special tests (such as fluorescence *in situ* hybridization, immunofixation electrophoresis, and chromosomal karyotype) performed according to clinical testing needs.

### Pathological morphology and immunohistochemistry

Bone marrow aspiration smears were prepared and stained with Wright-Giemsa stain. Bone marrow and lymphoid biopsies were prepared, fixed with formalin, embedded in paraffin, and stained with hematoxylin-eosin. Immunohistochemical analyses were performed on formalin-fixed, paraffin-embedded tissue using standard techniques with the antibodies required for actual clinical testing. Routine immunohistochemical staining of cluster of differentiation (CD) 19 and CD20 was performed, and additional stains included CD5, CD10, CD23, CD103, CD25, CD123, CD200, Ki-67, PAX-5, SOX-11, Cyclin-D1, BCL2, BCL6, and MYC according to clinical testing needs or doctors’ orders.

### Flow cytometry

Flow cytometry (FC) was performed on fresh bone marrow aspiration and/or peripheral blood samples. The lymphoma-associated cell surface markers CD19, CD20, CD5, CD10, Kappa, and Lambda were routinely examined, while the markers FMC7, CD22, CD23, CD25, CD38, CD138, CD103, CD200, and IgM were examined according to clinical testing needs or doctors’ orders based on five-color analyses (FITC, PE, ECD, PC5, and PC7). FC was performed on Cytomics FC500 Cytometer (Beckman Coulter, Brea, CA, USA), and the data were analyzed with FCS Express flow cytometry software (*De Novo* Software, Los Angeles, CA, USA).

### Next-generation sequencing and variant curation

An NGS panel consisting of 175 genes associated with hematological malignancy (175-Panel) was applied for all 849 patients in this study (**Supplement Table 1**). A QIAamp DNA Mini Kit (Qiagen, Hilden, Germany) was used for DNA extraction from formalin-fixed paraffin-embedded lymphoid samples, bone marrow samples, and/or peripheral blood samples, and then a KAPA Library Amplification Kit (Kapa Biosystems, Wilmington, MA, USA) was used for library construction. DNA sequencing was performed on an Illumina NovaSeq6000 system (Illumina, San Diego, CA, USA) with DNA input of 500 ng on average and sequencing depth of 1000X on average. Variant calling was performed with the Somatic Variant Caller Algorithm from Illumina with default filtering settings. The sequencing data were included if meeting the following minimum quality control (QC) standards: 50X coverage of target region ≥99%; average sequencing depth ≥200X; Q30 ≥0.85; and target region capture rate ≥99%. Variants were interpreted according to the Standards and Guidelines for the Interpretation and Reporting of Sequence Variants in Cancer (21). A variant was considered novel if it was absent in all of the following databases: COSMIC (22), dbSNP (23), ClinVar (24), gnomAD (25), ExAC (26), HGMD (27), 1000 Genomes (28), and ESP6500 (http://evs.gs.washington.edu/EVS/). Variants with strong clinical significance (Tier I) and variants with potential clinical significance (Tier II) were the focus of this study (21).

### Machine learning model construction

ML model construction data were collected from the Catalogue Of Somatic Mutations In Cancer (COSMIC) database (time range: database inception through May 20^th^, 2021) (22). Variant interpreting procedures were followed according to the same standard used by our laboratory. The data were divided into a training dataset and a test dataset with a proportion of 8:2 by stratified sampling. In total, eight ML algorithms, namely, Random Forest (RF), K-Nearest Neighbors, Naive Bayes, Recursive Partitioning, Neural Network, Gradient Boosting Machine, Logic Regression, and Support Vector Machines, were used for the pretest of ML model construction in this study (**Supplement Figure 1**). The m×n (the number of cases from ML datasets × the number of ML model features) data matrix was designed for ML model construction. ML model features included NGS-related and tumor cell size-related features (**Supplement Table 2**). Concerning NGS-related ML model features, mutated genes with Tier I and/or Tier II variants were marked as “1 (representing positive by metric variable)” whereas mutated genes without Tier I and/or Tier II variants and unmutated genes as “0 (representing negative by metric variable)” in the matrix. Similarly, tumor cell size was categorized as small to medium or medium to large, and the cases with small to medium tumor cell size were marked as “1” while those with medium to large as “0”. The tumor cell sizes of the cases in the COSMIC database were broadly represented according to their pathological subtypes. Five repeats of 10-fold cross-validation were conducted in the training and internal validation sets. Model feature selection was based on the method of Recursive Feature Elimination (RFE). The ML models, COSMIC I (COSMIC IA and IB) and COSMIC II (COSMIC IIA and IIB), were constructed in this study. COSMIC I (COSMIC IA and IB) were constructed based only on NGS results, and COSMIC II (COSMIC IIA and IIB) were constructed based on combining NGS results and tumor cell size. COSMIC IB and COSMIC IIB were obtained with the highest model efficiency after model feature selection of COSMIC IA and COSMIC IIA, respectively. Model efficiency was defined as the model that achieved the higher accuracy with the lower number of genes, and five gradient levels (95~99% of the highest model accuracy) were used to compare this indicator. Base learners were selected by considering accuracy (95% CI) and kappa for each diagnostic class in the validation set.

### Statistical analyses

Statistical analyses were performed using R version 4.1.0. ML model construction was performed using the R software package “caret”. Mutation landscape analyses were performed using the R software package “maftools” and viewed with the R software package “trackViewer”. Patient groups were evaluated by using the χ^2^ test or Fisher’s exact test. *P* values<0.05 were considered statistically significant.

## Results

### Patient summary and mutation landscape

**Figure 1:**
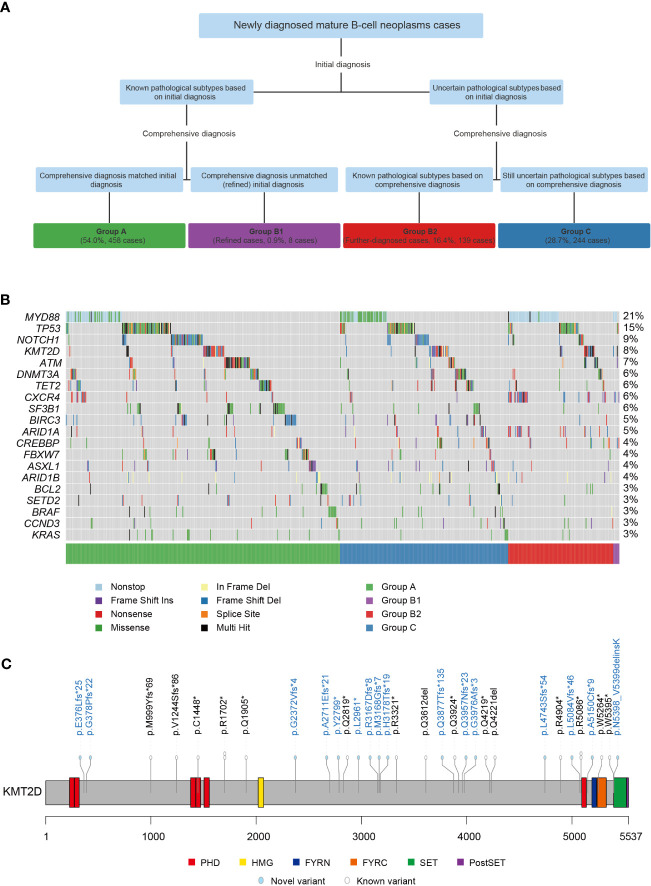
Grouping procedure and mutation landscape of 849 cases of mature B-cell neoplasms (MBNs). **(A)** Four groups of 849 MBN cases subcategorized into initial diagnosis and comprehensive diagnosis. **(B)** The mutation landscape of the top 20 genes detected in 849 MBN cases. **(C)** Localization and frequencies of 33 *KMT2D* variants in 26 CLL/SLL cases. *: Stop codon.

A total of 849 cases of MBNs were included in this study. The subtype was identified at initial diagnosis in 458 cases, which was also the same as their comprehensive diagnosis (54.0%, Group A). When considering multiple platforms from the MICM classification system, 139 cases with uncertain subtypes at initial diagnosis were further identified by comprehensive diagnosis (further-diagnosed cases, 16.4%, Group B2). Interestingly, 8 cases were different between the initial diagnosis and comprehensive diagnosis, which suggests that misdiagnosis existed in the initial diagnosis (refined cases, 0.9%, Group B1). However, there were still 244 cases with an uncertain subtype (28.7%, Group C) (**Figure 1A**). In summary, 9 subtypes of MBNs, namely, Burkitt lymphoma (BL), CLL/SLL, DLBCL, FL, hairy cell leukemia (HCL), high-grade B-cell lymphoma (HGBL), LPL/WM, MCL, and marginal zone B-cell lymphoma (MZBL), were found to be involved.

Overall, 1850 variants present in 107 (61.1%) of the 175 genes were detected in 690 (81.3%) of the 849 MBN cases (**Supplement Table 1**). Among these genes, *MYD88* (20.8%), *TP53* (14.6%), *NOTCH1* (8.6%), *KMT2D* (8.5%), and *ATM* (6.7%) were the most recurrently mutated genes in MBNs in our cohort (**Figure 1B**). The mutation landscape of each subtype is summarized in **Supplement Table 3**. Significantly, CLL/SLL showed some differences while other subtypes were essentially consistent with previous studies (6–17). *KMT2D* was rarely mutated in CLL/SLL according to previous studies in Western countries (6–8), but 33 variants in 26 (6.9%) cases of CLL/SLL were found in this study (one case harbored 5 *KMT2D* variants and three cases harbored 2 *KMT2D* variants) (**Figure 1C**). In addition, 544 (29.4%) novel variants were identified and are shown in **Supplement Figure 2**, and the detailed results are listed in **Supplement Table 4**.

### Machine learning model construction based on the COSMIC database

Model training datasets with large sample sizes are essential for model construction. Here, we used MBN cases from the COSMIC database to construct ML models to assist with differential diagnosis. In total, 8895 cases of 8 MBN subtypes (BL, CLL/SLL, DLBCL, FL, HCL, LPL/WM, MCL, and MZBL) were collected from the COSMIC database for model construction (**Supplement Table 5**). Eight ML algorithms were used for the model construction pretest, and ultimately, RF was selected for subsequent ML model construction on the basis of its high accuracy in the pretest and its proven effectiveness and popularity in previous studies (29, 30). Detailed results of the ML model construction pretest are shown in **Supplement Figure 1** and **Supplement Table 6**.

Next, we constructed ML models using only the NGS results for the 175-Panel genes (COSMIC IA), and the model accuracy was 0.74 (95% CI: 0.7211-0.7623; Kappa: 0.67) (**Supplement Table 7**). Interestingly, we found that a large proportion of incorrect predictions was due to poor discrimination between CLL/SLL and DLBCL according to NGS results but with clear differences in tumor cell size. To improve the model accuracy, based on the NGS results for the 175-Panel genes, we added tumor cell size to the model construction (COSMIC IIA). The model accuracy of COSMIC IIA was 0.88 (95% CI: 0.8587-0.8900; Kappa: 0.84) (**Supplement Table 7**).

The mutation status of the 175-Panel genes was used in the model construction of COSMIC IA and COSMIC IIA, but not every gene was of strong diagnostic significance in the subtype differential diagnosis of MBNs. Thus, we performed model feature importance analyses to identify the most effective features in these models. Finally, 104 genes with diagnostic significance (importance value>0) and only 32 genes with importance values greater than 10 were found in COSMIC IA, while 103 genes with diagnostic significance and only 24 genes with importance values greater than 10 were found in COSMIC IIA (**Supplement Table 2**).

**Figure 2:**
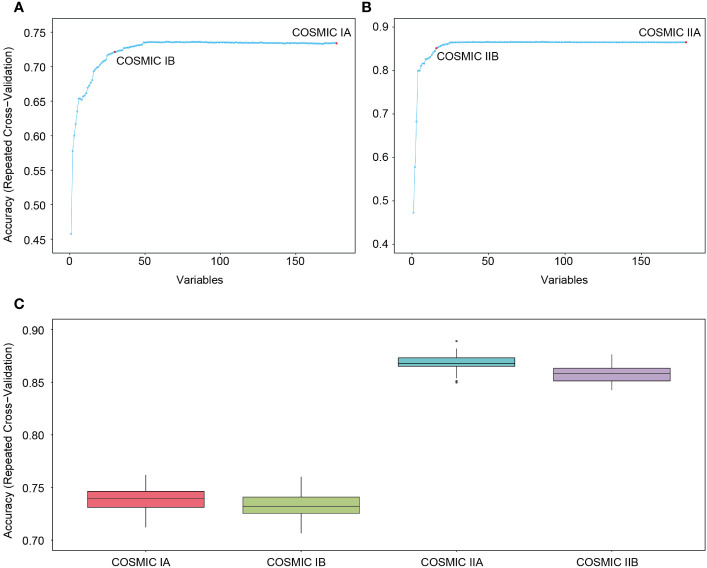
Construction and internal validation of machine learning (ML) models based on the COSMIC database. **(A)** Model feature selection in COSMIC IA. When the model feature number was 30 in COSMIC IA, the model had the highest efficiency (COSMIC IB). **(B)** Model feature selection in COSMIC IIA. When the model feature number was 16 in COSMIC IIA, the model had the highest efficiency (COSMIC IIB). **(C)** Model accuracy of COSMIC I (COSMIC IA and IB) and COSMIC II (COSMIC IIA and IIB) in internal validation.

By combining feature importance values, we further constructed more efficient ML models through feature selection analyses. The results showed that model accuracy improved with the increase in features in both COSMIC IA and COSMIC IIA (**Figures 2A, B**), and a model efficiency indicator of 98% was the suitable cut-off point in this study. Detailed results are shown in **Supplement Table 8**. Consequently, the models had the highest efficiency when the model feature number was 30 (30 genes) in COSMIC IA and 16 (14 genes and 2 tumor cell size features) in COSMIC IIA (**Figures 2A, B**). Thus, COSMIC IB and COSMIC IIB were obtained with the highest model efficiency after model feature selection (RFE, 98% cut-off) of COSMIC IA and COSMIC IIA, respectively. The model accuracies of COSMIC IB and COSMIC IIB were 0.73 (95% CI: 0.7119-0.7536; Kappa: 0.65) and 0.87 (95% CI: 0.8522-0.8842; Kappa: 0.83), respectively (**Figure 2C** and **Supplement Table 7**). Overall, we constructed four ML models at different levels using the COSMIC database, and COSMIC II (COSMIC IIA and IIB), based on integration of NGS testing and tumor cell sizes, showed superior effectiveness in the subtype classification of MBNs.

### Machine learning models predicted subtype diagnosis based on the local cohort

**Figure 3:**
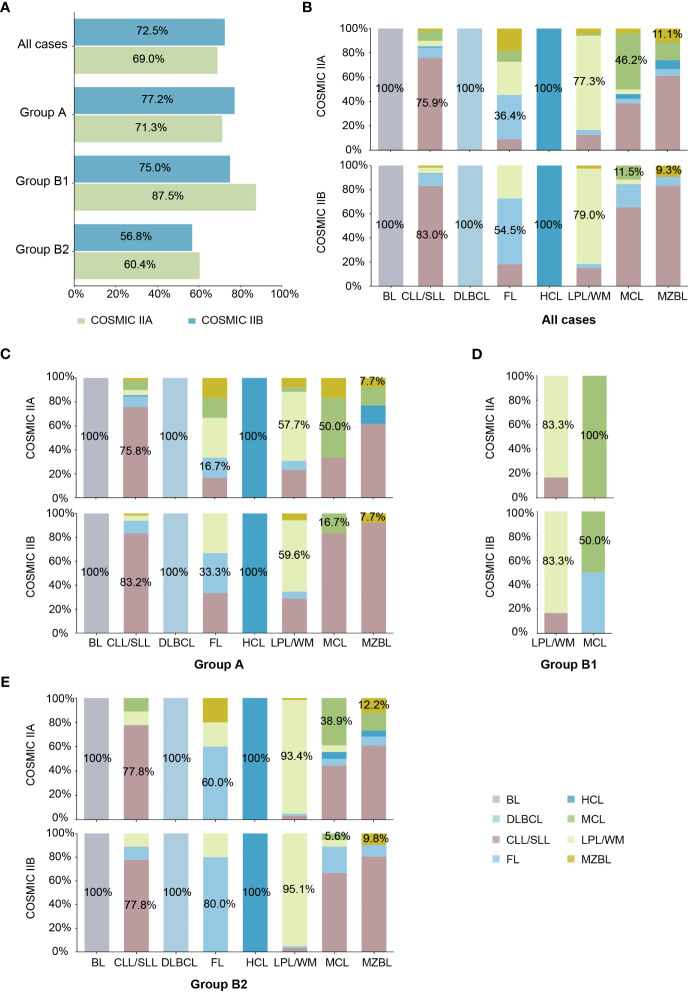
External validation of machine learning (ML) models based on local cohort. **(A)** Model accuracy of COSMIC II (COSMIC IIA and IIB) in each case group. **(B)** Model accuracy of COSMIC II (COSMIC IIA and IIB) by subtype in all cases. **(C)** Model accuracy of COSMIC II (COSMIC IIA and IIB) by subtype in typical cases (Group A). **(D)** Model accuracy of COSMIC II (COSMIC IIA and IIB) by subtype in refined cases (Group B1). **(E)** Model accuracy of COSMIC II (COSMIC IIA and IIB) by subtype in further-diagnosed cases (Group B2).

To investigate the clinical diagnostic performance of COSMIC II (COSMIC IIA and IIB) based on local patients, we used clinical cases from our laboratory to test their actual application. Known-subtype cases matching the eight subtypes of MBNs collected from the COSMIC database in this study (603 cases in Group A and Group B) were used for the next validation. Overall, the model accuracies of COSMIC IIA and COSMIC IIB for our cases were 0.69 and 0.73, respectively (**Figure 3A**). In terms of the pathological features, the ML models had the best prediction accuracy for cases in Group A with typical morphological and immunophenotype features; of the difficult cases in Group B, 75.0% in COSMIC IIA and 87.5% in COSMIC IIB of the refined cases (Group B1), and 56.8% in COSMIC IIA and 60.4% in COSMIC IIB of further-diagnosed cases (Group B2) were correctly predicted (**Figure 3A**). In terms of subtype, overall, the model performance was good in BL (accuracy of 100% in both COSMIC IIA and IIB), CLL/SLL (accuracy of 75.9% in COSMIC IIA and 83.0% in COSMIC IIB), DLBCL (accuracy of 100% in both COSMIC IIA and IIB), HCL (accuracy of 100% in both COSMIC IIA and IIB), and LPL/WM (accuracy of 77.3% in COSMIC IIA and 79.0% in COSMIC IIB), whereas it was not as effective in MCL (accuracy of 46.2% in COSMIC IIA and 11.5% in COSMIC IIB) and MZBL (accuracy of 11.1% in COSMIC IIA and 9.3% in COSMIC IIB) (**Figure 3B**). Detailed results of each group are shown in **Figures 3B–E**. In summary, using ML models to assist in morphological and immunological diagnosis demonstrated positive clinical potential in both groups and most subtypes of MBNs.

In addition, the MBN subtype classification models, COSMIC I (COSMIC IA and IB) and COSMIC II (COSMIC IIA and IIB), are available as web-based open-source resources that can be accessed widely by clinicians and the public to predict the subtype of MBNs (https://kingmed.shinyapps.io/cosmic_i/ and https://kingmed.shinyapps.io/cosmic_ii/).

### Analyses of the causes of incorrect model prediction results

**Figure 4:**
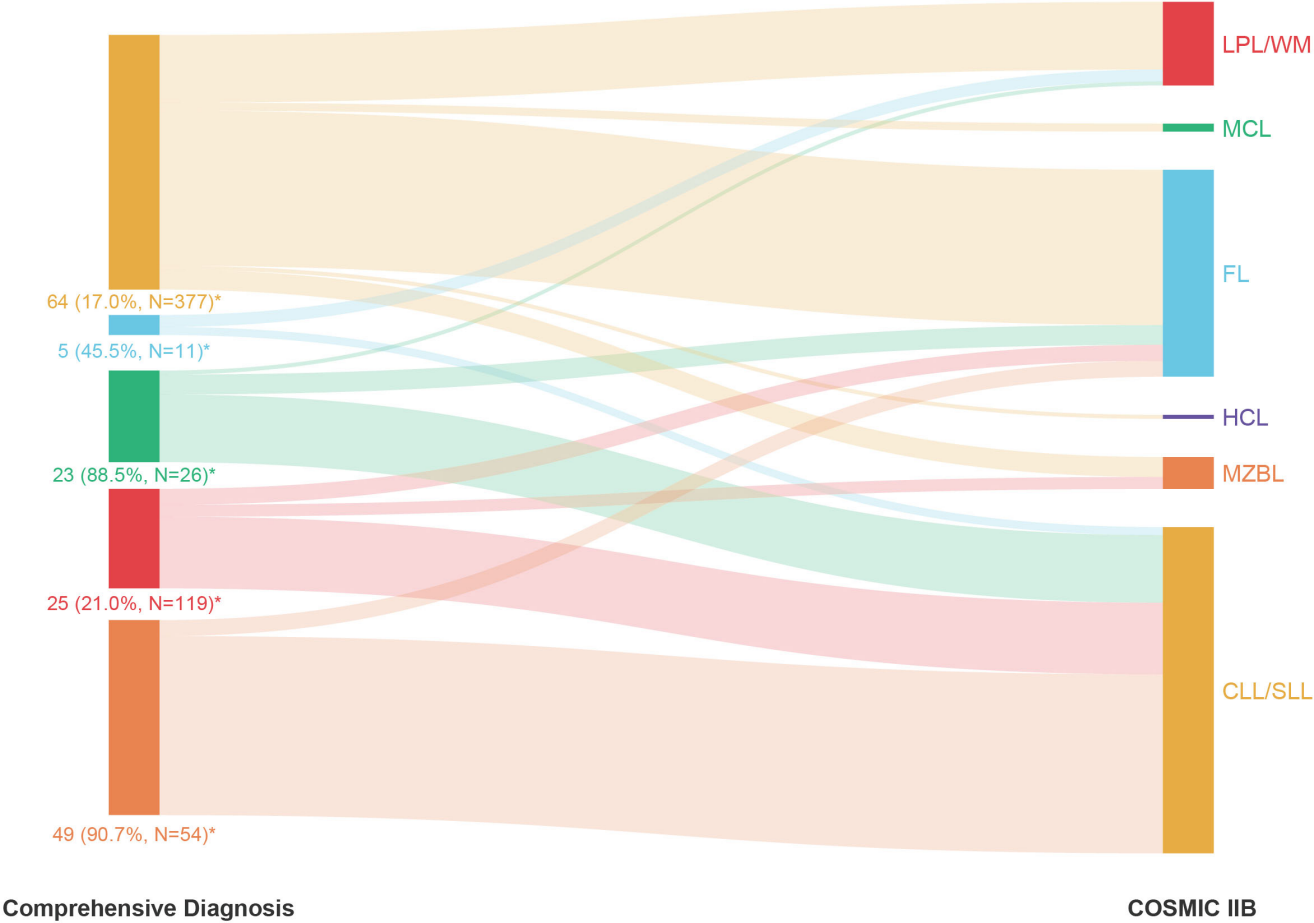
The proportion of cases incorrectly predicted by COSMIC IIB within each subtype of mature B-cell neoplasms (MBNs) based on comprehensive diagnosis. *: Number of incorrectly predicted cases (Incorrectly predicted rate, Total number of predicted cases).

We further summarized the incorrectly predicted cases of each subtype in **Supplement Table 7** and analyzed the reasons for incorrect model prediction results based on COSMIC IIB (**Figure 4**). First, 60.9% (39/64) of CLL/SLL cases that were incorrectly predicted as FL harbored *KMT2D* variants, which are considered relatively specific molecular characteristics of FL in Western populations but highly recurrent in Chinese CLL/SLL. Thus, population differences were one of the possible reasons for incorrect model predictions, suggesting that clinicians should pay attention to ethnicity when interpreting NGS results for subtype differential diagnosis. Second, 92% (23/25) of all incorrectly predicted LPL/WM cases had no *MYD88* L265P or *CXCR4* variants, which are considered specific molecular characteristics in LPL/WM and helpful for the differential diagnosis of LPL/WM, indicating that an atypical mutation landscape was another possible reason for incorrect model predictions. Such atypical cases need more support from other special platforms, such as immunofixation electrophoresis, in differential diagnosis clinically. Third, there were some cases harboring variants specific to other subtypes, such as *BRAF* V600E in CLL/SLL, which caused these cases to be more likely to be misdiagnosed and made differential diagnosis more difficult, showing that the overlap of the mutation landscape among different subtypes was also a possible reason for incorrect model predictions. Consequently, although the case may have typical variants supporting the diagnosis of a certain subtype, other possible subtypes should also be considered and excluded. Finally, we noted that the size of the NGS panel also had some impact on incorrect model prediction, including the problems of over-consideration and incomplete-consideration. While 16 (2.7%) cases with incorrect predictions in COSMIC IIB were correctly predicted in COSMIC IIA, 37 (6.1%) cases with incorrect predictions in COSMIC IIA were correctly predicted in COSMIC IIB (**Supplement Table 7**). Over-consideration mainly occurred in CLL/SLL (accuracy of 75.9% in COSMIC IIA *vs*. 83.0% in COSMIC IIB), FL (accuracy of 36.4% in COSMIC IIA *vs*. 54.5% in COSMIC IIB), and LPL/WM (accuracy of 77.3% in COSMIC IIA *vs*. 79.0% in COSMIC IIB), for which COSMIC IIB showed higher accuracy, while incomplete-consideration mainly occurred in MCL (accuracy of 46.2% in COSMIC IIA *vs*. 11.5% in COSMIC IIB) and MZBL (accuracy of 11.1% in COSMIC IIA *vs*. 9.3% in COSMIC IIB), for which COSMIC IIA showed better model performance (**Figure 3B**). Detailed results of each group are shown in **Figures 3B–E**.

## Discussion

NGS has been widely applied in routine MBN clinical detection, but its application in the differential diagnosis of MBNs is still uncertain (31, 32). This study retrospectively investigated 849 newly diagnosed cases of MBNs to investigate mutation landscapes in Chinese patients with MBNs and to combine mutational information and ML into clinical applications for MBNs, especially for subtype classification.

Based on the cohort of newly diagnosed MBNs from our laboratory, two diagnostic groups, namely, initial diagnosis and comprehensive diagnosis, were defined to investigate the current status of the clinical diagnosis of MBNs. Conventional morphology and immunology testing remained the primary and necessary platform for the differential diagnosis of MBNs and were capable of diagnosing the majority of cases (54.0%) with typical pathological features. In addition, a subset of cases (17.3%) required multiple platform testing to complement and confirm the initial pathological diagnosis. However, there was still a notable proportion of cases (28.7%) in which a definitive diagnosis of the MBN subtype was not obtained through the comprehensive diagnostic system, suggesting major clinical challenges in the differential diagnosis of MBNs and the need to develop adjunctive assisting diagnostic methods based on existing NGS testing platform.

The combined application of NGS and ML in the differential diagnosis of MBNs is still uncertain. To explore a suitable ML model for the differential diagnosis of MBNs, a series of ML models were constructed. Among these models, COSMIC IIB had the best efficiency and greatest model accuracy, and its model features were well represented. Tumor cell size distinguished the subtype of small B-cell lymphomas and large B-cell lymphomas. In small B-cell lymphomas, *MYD88* L265P and *BRAF* V600E are recognized as typical variants of LPL/WM and HCL, respectively (16, 33). *CXCR4* is another commonly mutated gene in LPL/WM (17, 34). *NOTCH1* variants and *SF3B1* variants are highly enriched in CLL/SLL (7, 8). Variants of *KMT2D*, *CREBBP*, and *BCL2* have emerged as hallmarks of FL (35, 36). Significantly, *KMT2D* variants have been rarely reported in CLL/SLL in previous Western studies but showed a high occurrence in this study, consistent with another Chinese study (37, 38), indicating that *KMT2D* variants are probably unique molecular characteristics in the subset of Chinese patients with CLL/SLL. Variants of *NOTCH2* and *TNFAIP3* are characteristic of MZBL (39, 40). *CCND1* variants have been identified recurrently in MCL (14, 15). In large B-cell lymphomas, *MYC* and *ID3* are recurrently mutated in BL, while *MYD88* L265P is commonly observed in DLBCL (9, 41, 42). These genes used for differential diagnosis in ML models were consistent with clinical findings and basic research, demonstrating the validity of the ML models.

To evaluate the application of ML models in diverse clinical situations, we designed corresponding case groups in this study. The best predicting accuracy was observed in typical cases (Group A) with ML models, indicating that cases with typical morphological features generally had typical molecular abnormalities, and consequently, the NGS results and model prediction results were highly consistent with the multiple platform testing results (2). The cases in Group B were difficult cases, including refined cases (Group B1) and further-diagnosed cases (Group B2). A series of refined cases were correctly predicted, valuable for reminding clinicians of the possibility of misdiagnosis, and large numbers of further-diagnosed cases were correctly predicted, which could be useful in differential diagnosis when the initial diagnosis is uncertain. The ML models confirmed the diagnosis of typical cases and suggested a potential subtype diagnosis for difficult cases.

Nevertheless, our study has multiple limitations that must be carefully considered. First, due to the lack of a large cancer database based on the Chinese population, using the COSMIC database, which mainly represents Western populations, to construct the ML model may underestimate the population diversity associated with genetic background. In addition, incorrect prediction cases with obvious mutational characteristics could be analyzed for the possible reasons for their incorrectly predicted, but there were still many cases that could not be analyzed due to the complexity of ML. The incorrectly predicted cases influenced by the size of the NGS panel should also be considered seriously. However, we still obtained excellent prediction results, and multiple strategies can be applied to improve these limitations in future research — the utility of extensive local databases can address genetic background bias while the addition of more testing platforms is vital for the interpretation of model prediction results and the improvement of model accuracy. As the local cancer database becomes increasingly larger with more comprehensive collections of clinical testing platform results, we believe that the model based on the local cancer database will play a greater role in the differential diagnosis of MBNs.

In conclusion, this study applied NGS to clinical practice *via* ML-assisted differential diagnosis of MBNs, and the ML models showed great significance at various levels. Despite several problems, NGS still shows a great deal of potential as an independent additional diagnostic tool for the clinical diagnosis process, especially for some special subtypes and difficult cases. With an increasingly accurate and comprehensive mutation landscape of MBN cases reported and combined with more platform results, the application of NGS in clinical diagnosis will be increasingly extensive and useful.

## Data availability statement

The datasets presented in this study can be found in online repositories. The names of the repository/repositories and accession number(s) can be found below: https://ngdc.cncb.ac.cn/, GVM000395.

## Ethics statement

The studies involving human participants were reviewed and approved by Medical Research Ethics Committee of the Third Affiliated Hospital of Sun Yat-sen University. Written informed consent from the participants’ legal guardian/next of kin was not required to participate in this study in accordance with the national legislation and the institutional requirements. Written informed consent was obtained from the minor(s)’ legal guardian/next of kin for the publication of any potentially identifiable images or data included in this article.

## Author contributions

LL, SY, YFM, YC and YHM designed the study. YFM, YC and YHM analyzed and interpreted the data. TC, XF, JL, JY, JP, GL and YL performed the related experiments and contributed to technical support. JF and KD performed variants curation. YFM wrote the manuscript. LL and SY conducted study supervision. This manuscript is approved by all authors for publication.

## Supplementary material

Supplementary Figure 1 Procedures of the machine learning (ML) model construction pretest.

Supplementary Figure 2 Novel variants in 849 cases of mature B-cell neoplasms (MBNs) cases. **(A)** The proportion of novel variants and known variants in different mutation types. **(B)** The proportion of novel variants in each subtype of MBNs. **(C)** The proportion of novel variants and known variants in the 15 most recurrently mutated genes.
