# Supplementary figures and images for: Machine learning models-based on integration of next-generation sequencing testing and tumor cell sizes improve subtype classification of mature B-cell neoplasms
Source: Front Oncol. 2023 Aug 3;13:1160383. doi: 10.3389/fonc.2023.1160383 (PMC10436202; doi:10.3389/fonc.2023.1160383)

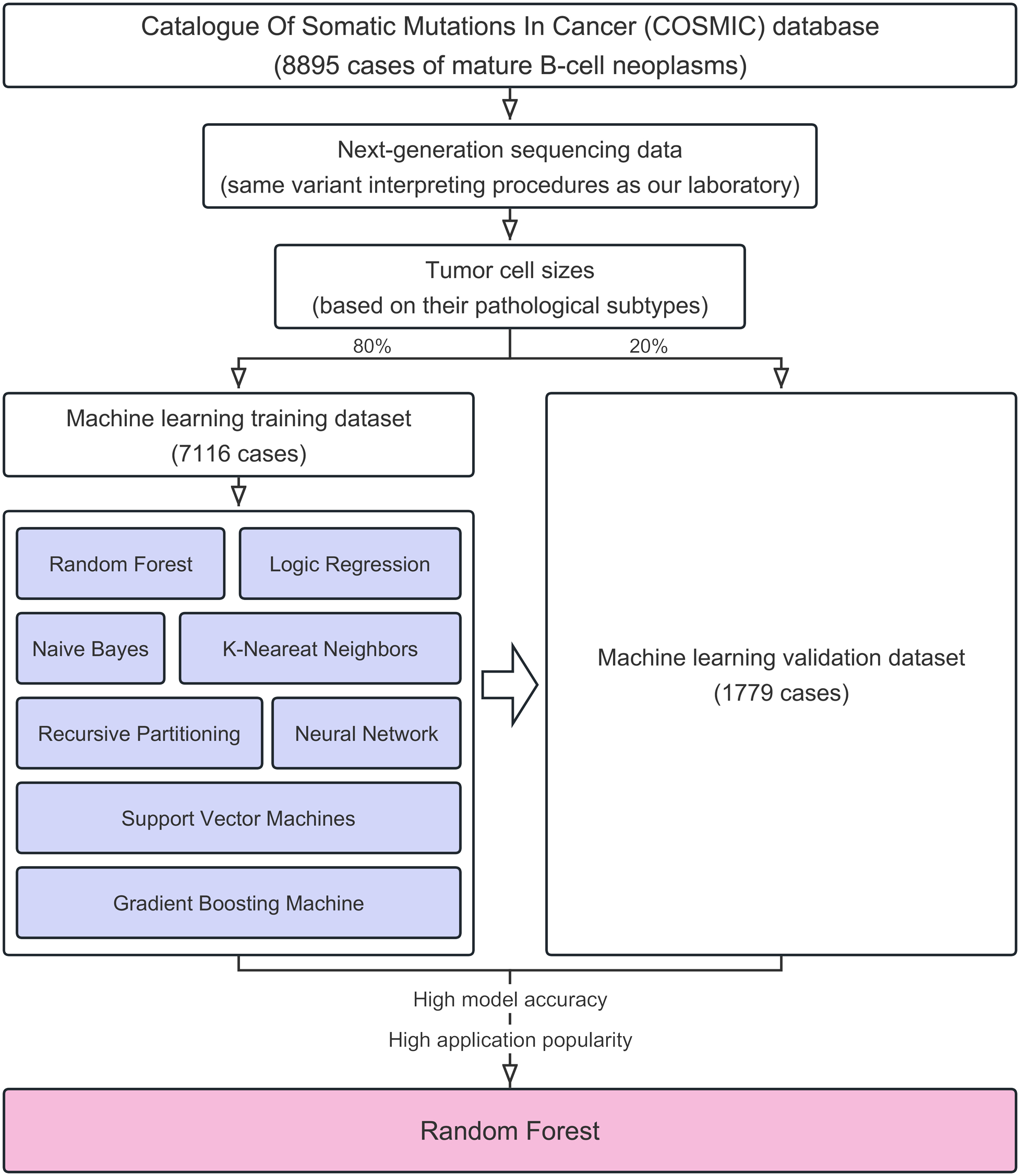

Supplement: Supplementary Figure 1 — Procedures of the machine learning (ML) model construction pretest. [file Image_1.tif]

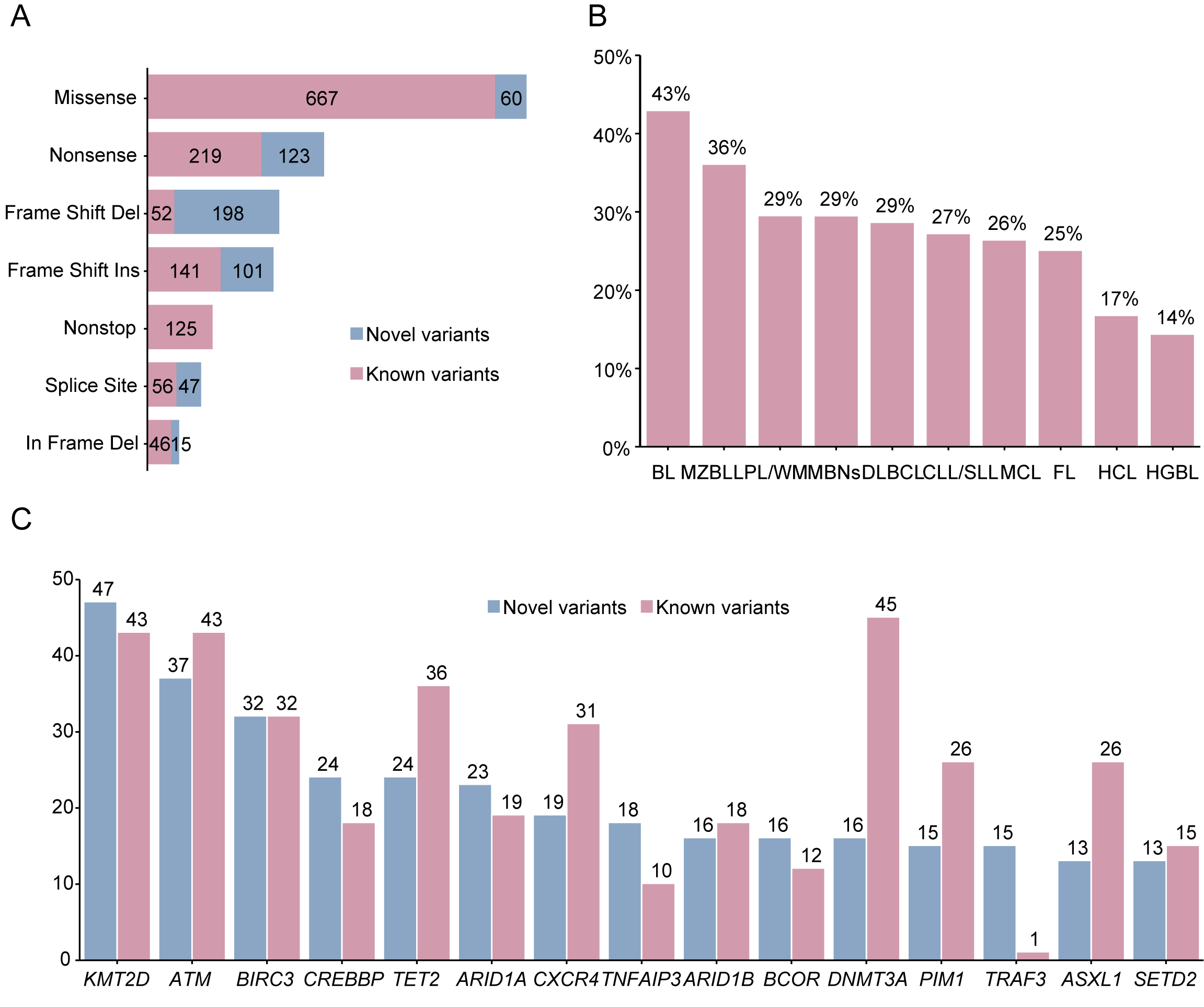

Supplement: Supplementary Figure 2 — Novel variants in 849 cases of mature B-cell neoplasms (MBNs) cases. (A) The proportion of novel variants and known variants in different mutation types. (B) The proportion of novel variants in each subtype of MBNs. (C) The proportion of novel variants and known variants in the 15 most recurrently mutated genes. [file Image_2.tif]
